# Racism and Cumulative Dis/Advantage in Healthcare Access: Implications for the Life Course

**DOI:** 10.1093/geroni/igaa057.1958

**Published:** 2020-12-16

**Authors:** Dale Dannefer

**Affiliations:** CWRU, Cleveland, Ohio, United States

## Abstract

Despite its origins in the study of race in America in Gunnar Myrdal’s American Dilemma, research on cumulative dis/advantage (CDA) and the life course has paid little attention to the significance of racism in the overall production and patterning of CDA. Building on recent work that has reviewed the life-course implications of the inscribing of racist interests in social policy, this paper explores the life-course implications of race bias in another domain, specifically the domain of medical diagnosis, where algorithm formulas have been shown to disadvantage black patients based on economic and other parameters. Even with training, experimental evidence comparing human and AI diagnostics have demonstrated that despite improvements, residual racism is evident in differential diagnoses. We consider the life-course implications of this and similar race-based differentials in organizational decision-making as a component in systems of cumulating dis/advantage.

